# Predicting diet in brachyuran crabs using external morphology

**DOI:** 10.7717/peerj.15224

**Published:** 2023-04-10

**Authors:** Katia Quezada-Villa, Zachary J. Cannizzo, Jade Carver, Robert P. Dunn, Laura S. Fletcher, Matthew E. Kimball, Ainslee L. McMullin, Brenden Orocu, Bruce W. Pfirrmann, Emily Pinkston, Tanner C. Reese, Nanette Smith, Carter Stancil, Benjamin J. Toscano, Blaine D. Griffen

**Affiliations:** 1 Department of Biology, Brigham Young University, Provo, Utah, United States; 2 National Oceanic and Atmospheric Administration Office of National Marine Sanctuaries—National Marine Protected Areas Center, Washington DC, United States; 3 Baruch Marine Field Laboratory, University of South Carolina, Georgetown, South Carolina, United States; 4 North Inlet-Winyah Bay National Estuarine Research Reserve, Georgetown, South Carolina, United States; 5 Department of Biology, Trinity College, Hartford, Connecticut, United States

**Keywords:** Diet composition, Diet quality, Gut size, Morphological variation, Nonlethal methods, Percent herbivory, Trophic position

## Abstract

Morphological traits have often been used to predict diet and trophic position of species across many animal groups. Variation in gut size of closely related animals is known to be a good predictor of dietary habits. Species that are more herbivorous or that persist on low-quality diets often have larger stomachs than their carnivorous counterparts. This same pattern exists in crabs and in most species, individuals exhibit external markings on the dorsal side of their carapace that appear to align with the position and size of their gut. We hypothesized that these external markings could be used as an accurate estimate of the crab’s cardiac stomach size, allowing an approximation of crab dietary strategies without the need to sacrifice and dissect individual animals. We used literature values for mean diet and standardized external gut size markings taken from crab photographs across 50 species to show that percent herbivory in the diet increases non-linearly across species of brachyuran crab with the external estimate of gut size. We also used data from dissections in four species to show that external gut markings were positively correlated with gut sizes, though the strength of this correlation differed across species. We conclude that when rough approximations of diet quality such as percent herbivory will suffice, measuring external carapace markings in crabs presents a quick, free, non-lethal alternative to dissections. Our results also provide important insights into tradeoffs that occur in crab morphology and have implications for crab evolution.

## Introduction

Ecomorphological studies typically focus on relationships between an organism’s morphology and its environment at fine spatial scales (Losos, Warheitt & Schoener, 1997). In addition, morphology varies both within and across species, and this variation can increase a species’ ability to occupy different habitats, persist in uncertain environments, and stabilize its interactions with other species (Noel et al., 2021; Toscano et al., 2022). Morphological variation therefore provides insight into the abilities and the strategies of organisms for performing daily tasks (Wainwright, 1991), and how these abilities and strategies differ both between and within species. Thus, the study of morphology can provide abundant insight into important ecological questions.

Food webs are a central part of ecological systems and extensive efforts have been made to elucidate dietary strategies and understand trophic links in terrestrial, freshwater, and marine systems. A key aspect of this is diet reconstruction, which is often conducted through a visual evaluation of gut content (*e.g*., Fincel et al., 2014; Guerra-García et al., 2014), the analysis of stable isotopes in the tissues of the consumer and its potential resources (*e.g*., Post, 2002), DNA barcoding of stomach contents (*e.g*., Taylor et al., 2022), or other molecular or chemical approaches (Nielsen et al., 2018). However, these techniques are not without drawbacks. Visual gut content analysis can underestimate soft-bodied prey due to the rapid digestion rate of these foods and because of the difficulty of accurately identifying prey without hard calcified structures (Koester & Gergs, 2017). Similarly, stable isotope approaches also have shortcomings since values are a product of trophic interactions as well as various biological and chemical processes (*e.g*., digestion, assimilation, fractionation), yielding an indirect characterization of diet (Layman et al., 2011). Additionally, given the indirect nature of the data and long time scales reflected (*i.e*., months), results can often be difficult to interpret and typically reflect broad characterizations of trophic relationships (Layman et al., 2011). Finally, while useful, DNA barcoding, is expensive and is limited by the availability of a reference database of genetic codes for possible food sources.

Morphological traits have often been used to predict the diet and trophic position of species across many animal groups, with varying levels of success. For example, measurements of mouth size, as well as gill raker number and length, can be used to make accurate diet estimates in many species of fish (Shoup & Hill, 1997; Snowberg, Hendrix & Bolnick, 2015). Similarly, measurements of individual mandibular depth, bill width, and skull length can be used to predict the trophic positions of birds (Corbin, Lowenberger & Gray, 2014). Skull and head size have also been used to make dietary predictions for both rodents and insects (Gibb et al., 2015; Verde Arregoitia, Fisher & Schweizer, 2017). Other commonly used morphological measurements applied to predict prey preference and foraging efficiency include teeth shape and size, appendage length, and general body size (Brigham & Fenton, 1991; Barton et al., 2011; Verde Arregoitia, Fisher & Schweizer, 2017). In crustaceans, claw morphology (length, height, and width) and closing force can be used to predict diet, prey selection, and foraging efficiency (Yamada & Boulding, 1998; Silva et al., 2010, 2017).

Gut size represents another morphological metric for assessing diet, based on the theoretical expectation that gut size should be inversely related to diet quality because organisms that eat low quality foods must eat large volumes in order to meet metabolic needs (Sibly, 1981). While not universally supported (*e.g*., mammalian herbivorous fermenters: Hume, 1989), this expectation has been supported across a broad range of taxa, including mammals (Borkowska, 1995), echinoderms (Foster & Hodgson, 1996), fish (Berumen, Pratchett & Goodman, 2011), and arthropods (Yang & Joern, 1994; Griffen & Mosblack, 2011). Supporting theoretical expectations, species that are more herbivorous or that persist on less labile or less nutritious diets typically have larger stomachs than their carnivorous counterparts. Consistent with these patterns, in brachyuran crabs, consumed food is first passed into the pyramid-shaped cardiac stomach, which increases in width and thus volume with percent herbivory in the diet (Griffen & Mosblack, 2011). In fact, gut width differences result in gut volumes for herbivorous species that are approximately 8× larger than the gut volumes of similarly sized carnivorous crab species (Griffen & Mosblack, 2011).

Differences in gut size not only occur across crab species with different diets, but also across individuals of the same species that use different diet resources. For instance, mangrove tree crabs (*Aratus pisonii*) are found in salt marshes, mangroves, and inhabiting anthropogenic boat docks within salt marshes. Crabs on boat docks consume higher quality diets that include more animal tissue than crabs in mangroves, and crabs in salt marshes have the lowest quality diet of all, based on lipid profiles (Cannizzo et al., 2020). As a result, gut sizes of crabs are smallest on docks, intermediate in mangroves, and largest in salt marshes (Cannizzo, Dixon & Griffen, 2018). Similarly, ghost crabs (*Ocypode quadrata*) consume less animal tissue and more algal material across beaches as human tourism levels increase, leading to strong increases in gut size with human tourism across these same beaches (Gül & Griffen, 2020). Finally, the invasive Asian shore crab (*Hemigrapsus sanguineus*) is found across New England sites that differ in community composition. At sites where animal foods are less available, crabs have larger guts, suggesting an increased reliance on lower quality foods such as algae (Griffen et al., 2020a). And a field experiment with this same species that enclosed individual crabs in cages containing both animal and algal foods found that individual crabs that chose to consume more algae during the 24-h experimental period had larger gut sizes after controlling for body size than crabs that chose to consume more animal tissue (Griffen & Mosblack, 2011). Thus, the size of the cardiac stomach increases, both across species and across individuals of the same species, in crabs that consume lower quality diets. It should also be noted that there are likely evolutionary constraints to stomach size in crabs due to the limited space inside inflexible exoskeletons (Hines, 1982).

Crabs are found in a wide range of marine, freshwater, and terrestrial habitats. They occupy a variety of trophic levels (Koch & Wolff, 2002; Nordhaus, Diele & Wolff, 2009; Vinagre et al., 2018) and therefore provide a pivotal link between primary production, detritus, and organisms at higher trophic levels, such as birds and fish (Sheaves & Molony, 2000; Bouillon, Koedam & Raman, 2002; Poon, Chan & Williams, 2009). Furthermore, the trophic interactions of this taxonomic group have conservation implications given that brachyuran crabs have been commonly introduced into coastal ecosystems worldwide (Galil, 2008). Thus, because there is considerable variation in the diet of crabs, assessing the dietary strategy of a given species is often necessary for ecological and conservation inquiry (Poon, Chan & Williams, 2009).

Diet in crabs is often assessed using gut content analysis (*e.g*., Giddins et al., 1986; Rudnick & Resh, 2005; Griffen, Guy & Buck, 2008; Bada et al., 2022), which requires dissection. Thus far, nonlethal diet estimates in crabs have been restricted to stable isotopes, which can be lethal depending on the tissue used, and claw morphology, which is generally only effective for carnivorous species (Yamada & Boulding, 1998; Potter, Cannizzo & Griffen, 2022). Developing non-destructive sampling methods is desirable for conservation and ethical reasons to help preserve individuals and populations (Awruch et al., 2008; Blaustein et al., 2017). In most crab species, individuals exhibit external markings on the dorsal side of their carapace that align with the position of their cardiac stomach (B. D. Griffen, 2011, personal observations). These markings can range from the simple curvature of the dorsal carapace in some species, creating a raised portion of the carapace in the region overlying the cardiac stomach, to lines and grooves along the dorsal carapace that align with the lateral margins of the cardiac stomach in other species. We hypothesized that these external markings could be used to accurately approximate the width of the crab’s cardiac stomach, which would allow us to make predictions about its dietary strategies (Griffen & Mosblack, 2011) without the need to sacrifice and dissect the animal. Measuring the width of these external markings could then be used as a nonlethal tool to better understand crab dietary strategies. Here we test the hypothesis that external carapace markings can be used to accurately assess the gut size, and therefore the diet, of brachyuran crabs. We do this both at the species level and at the individual level. At the species level we used crab pictures available from online databases and published diet strategies to examine mean changes in external gut markings with mean diet across 50 species. At the individual level, we examined four species of omnivorous, generalist crabs. Generalist populations are often composed of individual specialists that specialize on different food items (*e.g*., Woo et al., 2008; Vander Zanden et al., 2010). We therefore hypothesized that external gut markings would reflect individual variation in gut size within these populations, which previous work shows to reflect individual diet differences.

## Methods

### Interspecific variation

We examined external gut markings across crab species to determine whether the external markings were consistent with expectations based on the mean diet of individual species using diet data obtained for 50 crab species from the literature. Using Google Scholar searches for “brachyuran crab” and “diet”, we found studies that quantified the percent herbivory of various brachyuran crab species. Only studies where percent herbivory was reported, or where it could be determined based on data provided in the study, were selected. We made an effort to obtain data from a wide phylogenetic sampling of species, and for each study, we recorded the genus, species, geographic location of the study, and the percent herbivory in the diet (Table S1). However, it should be noted that our study only covers a small portion of the full phylogeny of brachyuran crabs.

For each species for which we had percent herbivory estimates, we used Google searches of the genus and species, as well as using common names, to locate photographs that were used to measure external gut markings. We chose photographs that clearly showed the dorsal carapace with sufficient clarity and lighting to enable measuring of the external gut markings. For each species, we confirmed the species identification using reliable species diversity websites, including the Crab Database (www.crabdatabase.info), the World Registry of Marine Species (www.marinespecies.org), and state and national species databases. Our aim was to use five photographs for each species; however, while this was possible for the majority of species (*n* = 37), this was not possible for all species given the number of quality photographs available (overall mean ± SD number of photographs used per species: 4.54 ± 0.93). The URL for each photograph used is given in Table S1.

**Figure 1:**
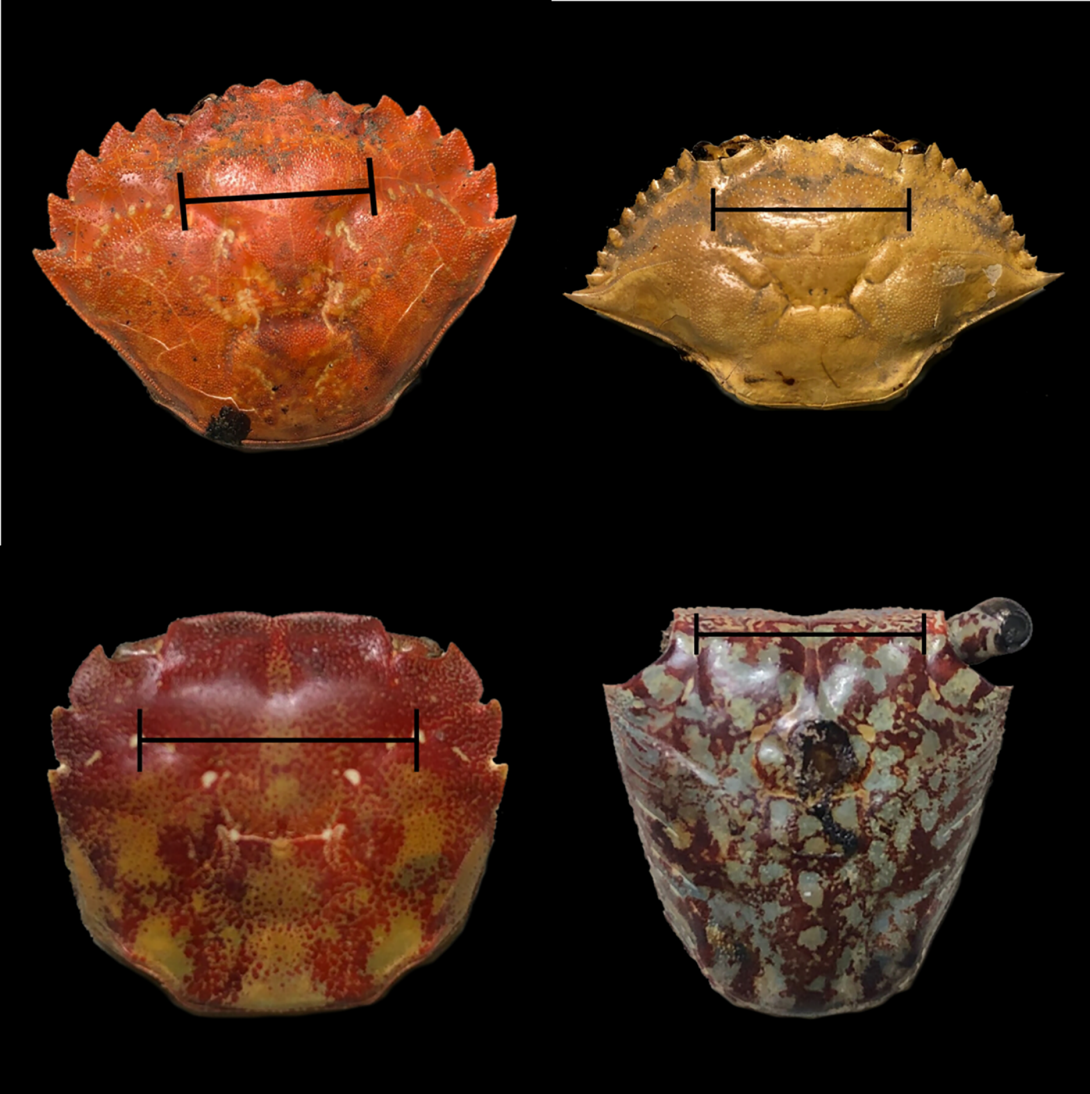
Carapace showing external gut markings (black lines) on each of the four species used for intraspecific comparison. External gut measurement done on each crab species (from left to right, top to bottom): European green crab *Carcinus maenas*, Atlantic blue crab *Callinectes sapidus*, Asian shore crab *Hemigrapsus sanguineus*, and mangrove tree crab *Aratus pisonii*. Black lines drawn over each carapace delineate the external gut markings as measured for these samples.

We used ImageJ (https://imagej.nih.gov/ij/) to measure the carapace width at the widest point (including the lateral spines), carapace length, the area of the carapace (by manually outlining the carapace and then using ImageJ to calculate the area outlined), and the width of the external gut markings from each photograph (as in Fig. 1). The scale of each photograph differed and was often not provided. Therefore, we used scale-independent metrics in our analysis. Specifically, we used the ratio of gut width:carapace width as a scale-independent measure of standardized gut width based on these external markings (Griffen & Mosblack, 2011). Data are not available on the relationship between gut width and carapace width for the majority of the species included in this study. However, using data on 15 brachyuran species from a previous study (Griffen & Mosblack, 2011), we verified that for all 15 species, gut width increases isometrically with carapace width. Thus, using the ratio of these two metrics for brachyuran crabs is not confounded by allometric growth. In addition, we multiplied the carapace width by carapace length to obtain the expected carapace area if the carapace had been a perfect square. We then divided this area into the observed area of the carapace to obtain a ratio that would approach 1.0 for a square-shaped carapace, but would decrease progressively below 1.0 the more the carapace diverged from a square. We label this quantitative metric of carapace shape the ‘carapace shape index’.

We used the mean values of standardized gut width and carapace shape index across each of the photographs for an individual species in the analyses described below. However, these two metrics were strongly positively correlated (see Results). Therefore, to avoid problems with collinearity, we used one of these metrics and the residual of the other metric after accounting for the first. To do this, we first regressed the carapace shape index against standardized gut width, and then used the residuals from this analysis (*i.e*., carapace shape after accounting for the impacts of standardized gut width) in the analysis described below. We also did the opposite, by regressing standardized gut width against carapace shape index and using the residuals from this analysis (*i.e*., standardized gut width after accounting for carapace shape) in the analysis. These two alternatives are identical in explaining the deviance in the data, but they yield complementary insights into understanding the relative importance of gut size and carapace shape, and so both are reported here. For each of the relationships described above, we examined the correlations using Pearson’s correlations after first confirming that the data were normally distributed using a Shapiro-Wilk test. Percent herbivory was not normally distributed, and so we used proportion herbivory and used an arc-sine square root transformation (W = 0.96, *P* = 0.08 after transformation) prior to conducting the Pearson’s correlations.

In both analyses, we used generalized linear mixed effects models (using the glmer function in the MASS package in R) to determine how percent herbivory in the diet (dependent variable) changed with either the mean standardized external gut marking width or with the carapace shape index, and with the residual of the other, measured on crab pictures collected from internet searches (fixed independent variables). Biologically, the choice of dependent and independent variables is likely opposite, as percent herbivory likely determines gut size and potentially carapace shape. However, we assigned the variables as described because our goal was to develop a tool that could be used to predict diet based on external estimates of standardized gut size. Since species differed slightly in the number of pictures available, we used the number of samples for each species as a weighting factor in the analysis. The size of the gut may be influenced by factors other than carapace shape that are correlated across phylogenetic relationships. We therefore explicitly controlled for phylogenetic relationship in our analysis by using Genus nested within Family as the random effect in our mixed effects model (de Villemereuil & Nakagawa, 2014; Griffen & Sipos, 2018). A significant effect of standardized external gut markings width in these analyses would therefore demonstrate the relationship between gut width and diet, even after accounting for phylogenetically driven differences in carapace shape or other phylogenetically-derived factors. Percent herbivory in the diet is inherently bounded between 0–100. We therefore converted values to proportions by dividing by 100 and then used a binomial error distribution in our generalized linear model.

### Intraspecific variation

We examined the ability to use external markings to assess the gut size, and thus the diet, of individual crabs using four different species of brachyuran crabs—the European green crab (*Carcinus maenas*), the Atlantic blue crab (*Callinectes sapidus*), the Asian shore crab (*Hemigrapsus sanguineus*), and the mangrove tree crab (*Aratus pisonii*). All four species are omnivorous, but the degree of omnivory varies among species (Griffen & Mosblack, 2011). The European green crab is an opportunistic, omnivorous predator, feeding on a wide variety of prey items (based on gut content analysis in Crothers, 1967; Baeta et al., 2006). Atlantic blue crabs are also opportunistic, omnivorous consumers in their native habitats, feeding on plants, invertebrates, and vertebrates (based on gut content analysis by Dittel, Epifanio & Fogel, 2006; Seitz, Knick & Westphal, 2011). The Asian shore crab is also an opportunistic omnivore that has substantial consumer impacts on its prey within the invaded North American area from which it was sampled (Lohrer & Whitlatch, 2002; Tyrrell, Guarino & Harris, 2006; Griffen & Byers, 2009). Diet selection studies show that Asian shore crabs feed on a variety of food resources including macroalgae and small invertebrates such as amphipods, gastropods, bivalves, barnacles, and polychaetes (McDermott, 1998; Brousseau, Filipowicz & Baglivo, 2001). Finally, mangrove crabs are an arboreal species that consumes primarily mangrove leaves, but will also readily consume animal prey (based on gut content analysis by Longonje & Raffaelli, 2014). For this study, we chose to test our hypothesis on individuals of these species because they are all omnivorous. We therefore expected diet and the resulting gut width to exhibit substantial variation within species. In addition, these species come from diverse habitat types, allowing us to apply our hypothesis broadly.

We focused on European green crabs, Atlantic blue crabs, Asian shore crabs, and mangrove tree crabs in our study, with all individuals collected as part of separate, earlier research efforts. Only females were collected for all but Atlantic blue crabs because earlier studies for which these crabs were collected only required females. Collection information for each species is provided in Table 1. All crabs were shipped frozen to Brigham Young University and were stored at −80 °C until processing. We measured the carapace width of each individual, as well as the external gut markings on each crab’s carapace to the nearest 0.01 mm using a digital caliper (Fig. 1). Atlantic blue crabs have a large lateral spine that can vary considerably in length, thus altering carapace width measurements. Inclusion of these lateral spines introduces additional variance into the data and therefore serves as a conservative test of our hypothesized relationships. We measured the carapace width both with and without this large spine (*i.e*., using the second spine), and the results did not qualitatively differ between the two measurements. We therefore only report results here for measurements that included the large lateral spines. Atlantic blue crabs also include adult and juvenile individuals. Maturity was determined based on the presence of discernible gonads during dissection. We dissected each crab by dorsal carapace removal. We extracted the crab’s cardiac stomach and measured the width of the cardiac stomach along the posterior ventral edge to the nearest 0.01 mm using a digital caliper. In addition, we also measured gut fullness on a scale of 0–4, with 0 being empty and 4 being completely full. Only crabs with guts at least half full (fullness of 2–4) were included in the analyses to ensure they maintained their shape.

**Table 1:** Collection information for species used in intraspecific comparisons.

Species	Collection location	Collection method	Size range (mm CW)	Number male	Number female	Number juveniles
*Carcinus maenas*	Rye, NH	By hand	27.7–63.3	0	65	0
*Callinectes sapidus*	Georgetown, SC	Seine net	14.6–168.1	42	20	90
*Hemigrapsus sanguineus*	Rye, NH	By hand	13.2–28.3	0	200	0
*Aratus pisonii*	Atlantic Intracoastal Waterway, FL	By hand	11.7–23.6	0	250	0

**Note:**

Collection information for each of the four species used here for examining intraspecific variation in external gut markings, including species, location, sampling methods, size ranges of crabs examined, and sample sizes for different demographics.

We analyzed the data independently for each of the four species, though similar methods were used for each. We first examined the relationship between actual gut width (dependent variable) and external gut width (independent variable) using regression analyses for each species. Next, gut size was expected to increase with crab body size, independent of individual diet, and our goal was to determine whether variation in gut width resulting from diet variation (*i.e*., independent of body size) could be determined from external gut markings. We removed the effects of body size by first regressing gut size on carapace width and external gut markings on carapace width. We then used the residuals from these two regression models in our analyses, regressing residual external gut markings (dependent variable) for each species against residual gut size (independent variable) (Zuur, Ieno & Smith, 2007). For the mangrove tree crabs, European green crabs, and Asian shore crabs we had only adult females and therefore conducted a single analysis for each of these species; however, for the Atlantic blue crab, we had adult males, adult females, and juveniles. Therefore, we analyzed the data for these three demographics separately. All statistical analyses were performed using R v. 3.6.0 (R Core Team, 2020).

This study did not require ethical approval. Animals were sacrificed by freezing in order to lower metabolic rates in a humane way and minimize suffering (Yue, 2008, Rotlant et al., 2023). We reduced the need for animal sacrifice in this study by using digital samples for interspecific comparisons, and by using samples that had been collected previously for other studies for each of the intraspecific comparisons.

## Results

### Interspecific variation

**Figure 2:**
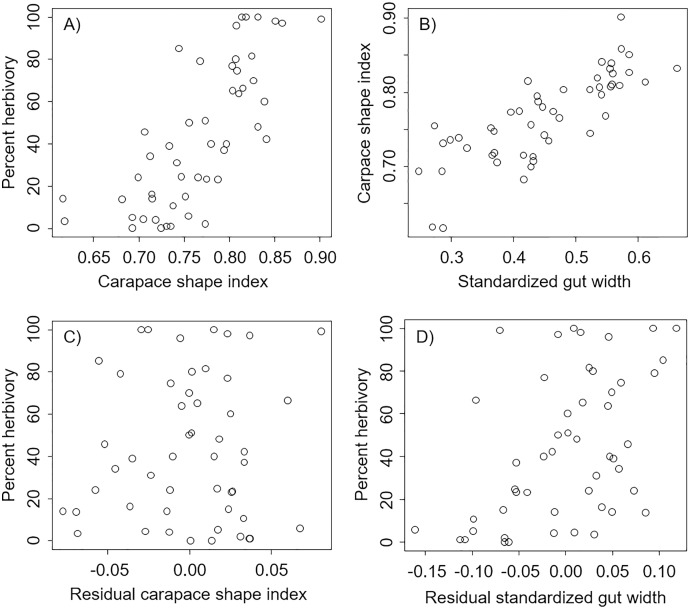
Correlations between metrics from each carapace picture in interspecific comparisons. Correlations across species between percent herbivory and the carapace shape index (A), between the carapace shape index and standardized gut width (B), between percent herbivory and residual carapace shape index after accounting for standardized gut width (C), and between percent herbivory and residual standardized gut width after accounting for carapace shape index (D).

We found that percent herbivory was strongly correlated with the shape of the carapace (Pearson’s correlation r = 0.74, *P* < 0.0001, Fig. 2A), and that the shape of the carapace was strongly correlated with the standardized external gut width (Pearson’s correlation r = 0.80, *P* < 0.0001, Fig. 2B). However, after accounting for the effect of standardized external gut width, there was no relation between the percent herbivory and residual carapace shape index (Pearson’s correlation r = 0.05, *P* = 0.70, Fig. 2C). In contrast, after accounting for the effects of carapace shape index, there was still a positive correlation between the percent herbivory and the residual standardized external gut width (Pearson’s correlation r = 0.49, *P* = 0.0003, Fig. 2D).

**Figure 3:**
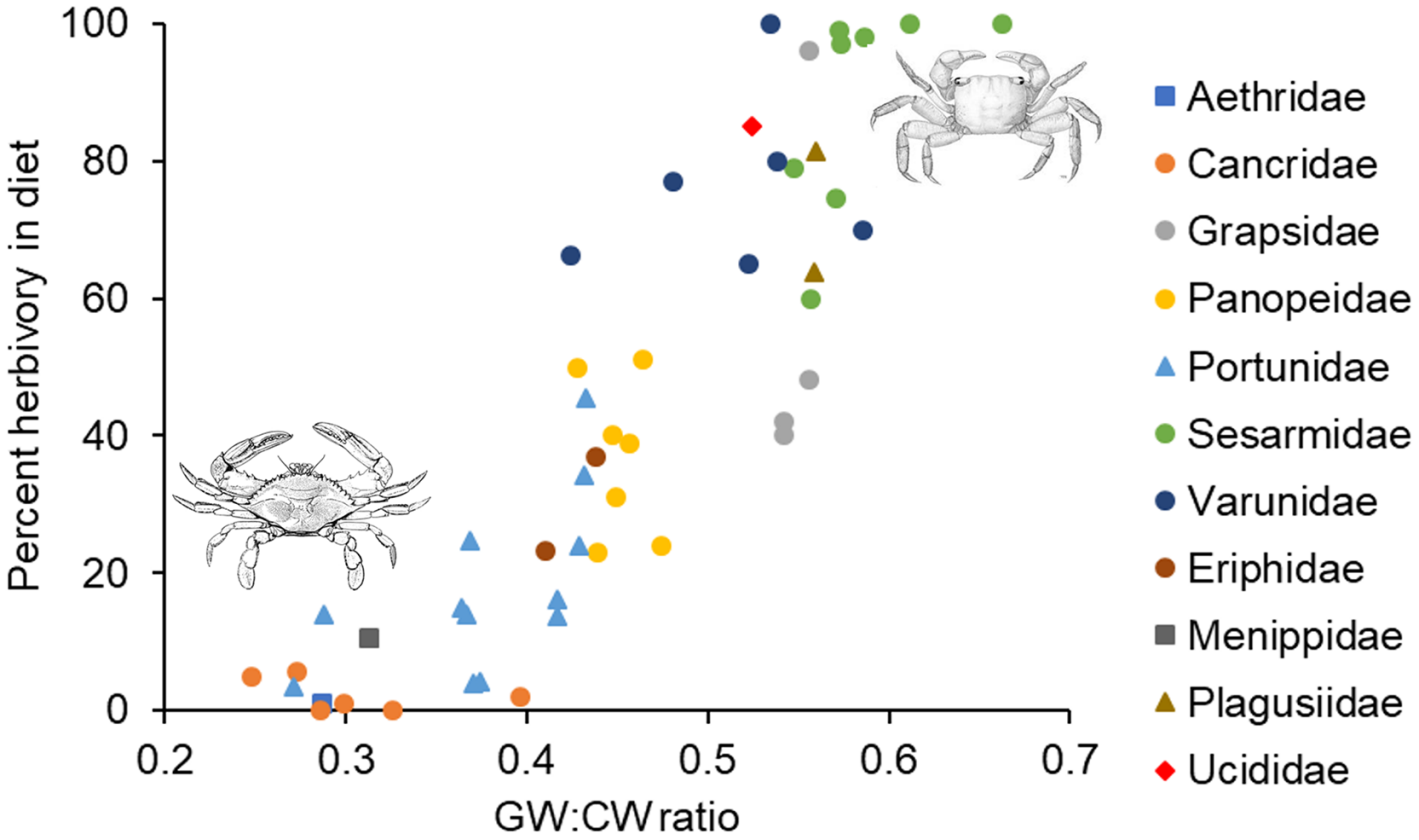
Relationship between percent herbivory in the diet and gut width to carapace width ratio across species. Interspecific relationship between arcsine square root transformed percent herbivory and the ratio of gut width (GW) to carapace width (CW), or the standardized gut width. Symbols represent the different phylogenetic families included in the analysis, as given along the right. Each data point shows the average value for the different species within a given genus. Crab drawing in upper right and lower left are, respectively, of *Sesarma reticulatum* and *Callinectes sapidus*, and are included to illustrate the differences in carapace shapes of crabs in these two different regions of the graph.

In the analysis that included standardized external gut size and residual shape index as fixed effects, a considerable amount of the variation in diet was explained by the phylogenetic relationship between species used in our analysis (*i.e*., by phylogenetically determined differences in the shape of the carapace or other phylogenetic factors), with most of this variation being explained at the Family level. Specifically, variance explained by phylogenetic Family was 7.07e−8 ± 2.66e−4, while variance explained by Genus nested within Family was 5.48e−8 ± 2.34e−4 (Fig. 3). Phylogenetic families differed in the extent to which they matched the expected positive relationship between percent herbivory and standardized gut width across genera within the same family. For instance, Portunidae, Sesarmidae, and Eriphidae matched the pattern strongly, while Cancridae and Varunidae did not. Even after accounting for phylogenetic relationships using these random effects, we found a clear increase in percent herbivory as the standardized gut size *via* external carapace markings increased (parameter estimate = 16.97 ± 2.21, *z* = 7.67, *P* < 0.0001, Fig. 3). However, there was no effect of shape index once the correlation with standardized gut width had been accounted for using residuals (*z* = 0.26, *P* = 0.79, Fig. 2C).

In the analysis that included carapace shape index and residual standardized external gut width as fixed effects, a considerable amount of the variation in diet was again explained by the phylogenetic relationship between species used in our analysis. Specifically, variance explained by phylogenetic Family was 1.65e−10 ± 1.29e−5, while variance explained by Genus nested within Family was 1.12e−10 ± 1.06e−5 (Fig. 3). In contrast to the analysis described above, we found a clear increase in percent herbivory with both the carapace shape index (parameter estimate = 12.60 ± 3.61, *z* = 6.81, *P* < 0.0001) and with residual standardized external gut width (parameter estimate = 16.37 ± 3.21, *z* = 5.10, *P* < 0.0001, Fig. 3).

### Intraspecific variation

For each of the species examined here, there was a strong correlation between the width of the external gut markings and the width of the actual gut. Specifically, gut width in Asian shore crabs increased by 0.67 ± 0.02 mm for each 1-mm increase in external gut width markings (*t* = 33.84, *P* < 0.0001, R^2^ = 0.85). Similarly, gut width in European green crabs increased by 0.48 ± 0.06 mm for each 1-mm increase in external gut width markings (*t* = 7.85, *P* < 0.0001, R^2^ = 0.70). Gut width in mangrove tree crabs increased by 0.73 ± 0.02 mm for each 1-mm increase in external gut width markings (*t* = 42.10, *P* < 0.0001, R^2^ = 0.88). Finally, gut width in Atlantic blue crabs increased by 0.52 ± 0.01 mm for each 1-mm increase in external gut width markings (*t* = 68.89, *P* < 0.0001, R^2^ = 0.95).

**Figure 4:**
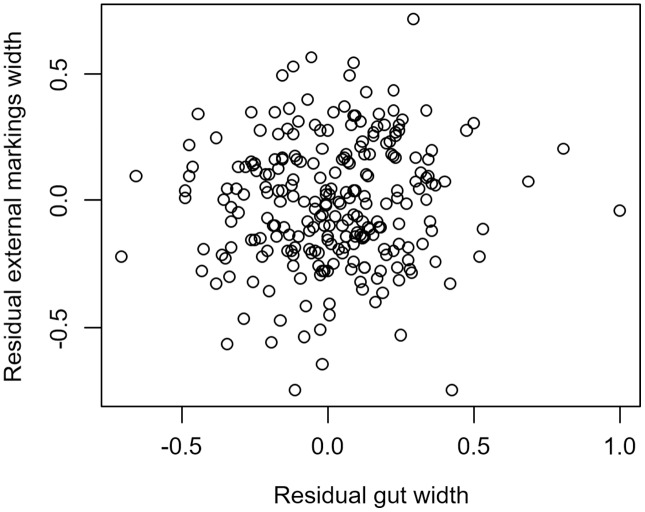
Residual external gut markings *vs*. residual gut width in *Aratus pisonii*. Relationship between residual external markings width and residual gut width (after accounting for body size) in the mangrove tree crab *Aratus pisonii*.

**Figure 5:**
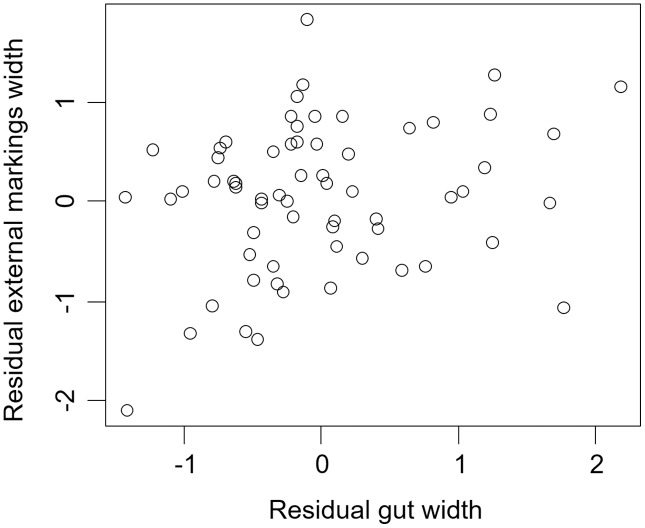
Residual external gut markings *vs*. residual gut width for *Carcinus maenas*. Relationship between residual external markings width and residual gut width (after accounting for body size) in the European green crab *Carcinus maenas*.

**Figure 6:**
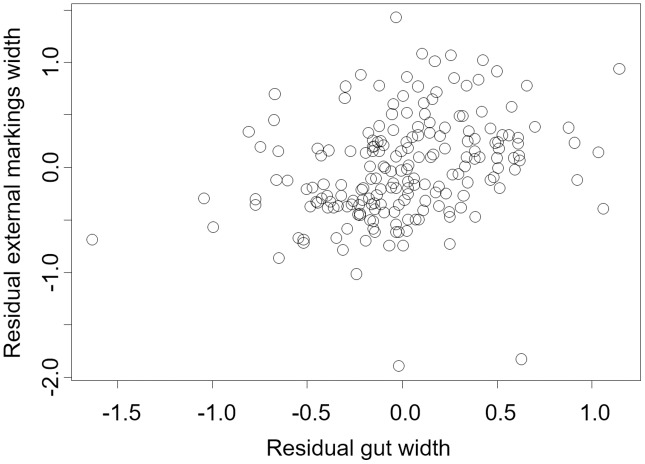
Residual external gut markings *vs*. residual gut width for *Hemigrapsus sanguineus*. Relationship between residual external markings width and residual gut width (after accounting for body size) in the Asian shore crab *Hemigrapsus sanguineus*.

**Figure 7:**
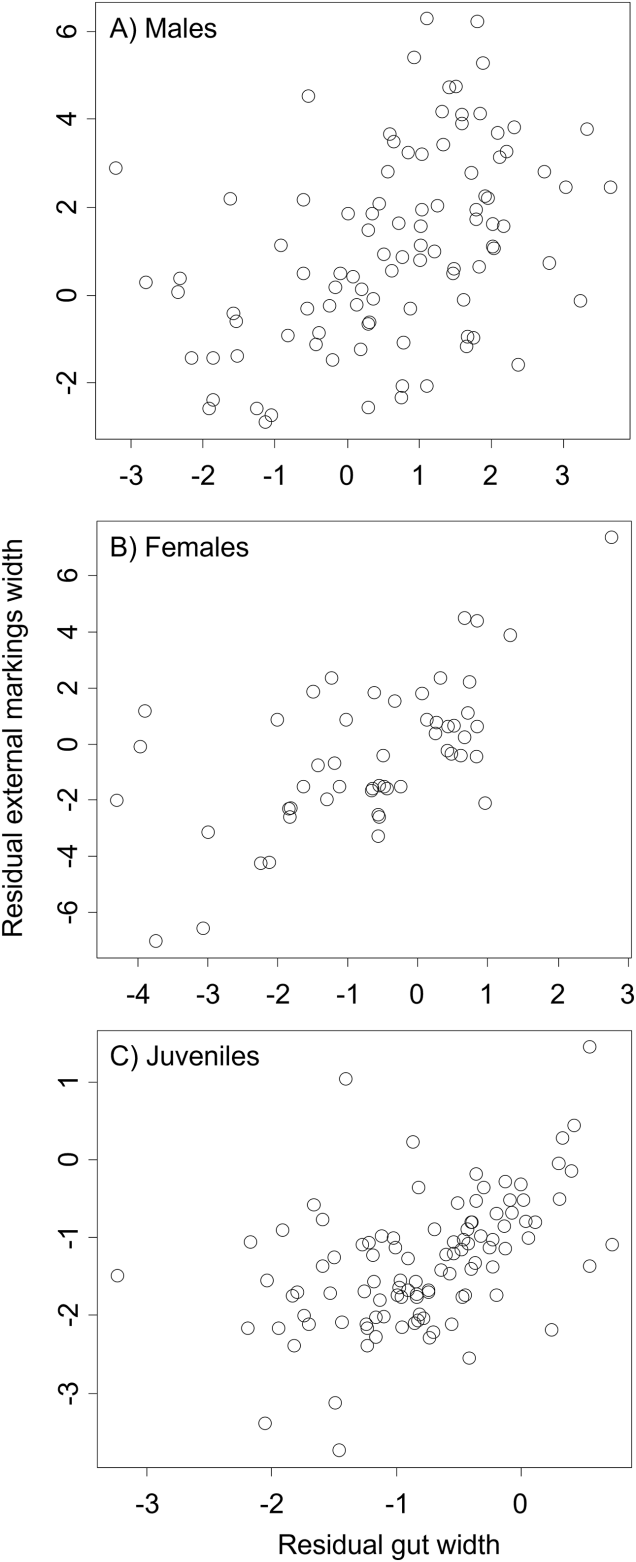
Residual external gut markings *vs*. residual gut width for *Callinectes sapidus*. Relationship between residual external markings width and residual gut width (after accounting for body size) in adult male (A), adult female (B), and juvenile (C) Atlantic blue crabs *Callinectes sapidus*.

We found that the relationship between variation in external gut markings and variation in the size of the gut, after controlling for differences in body size, differed across species. Specifically, residual external gut markings were unrelated to residual gut size in both the mangrove tree crab (*t* = 1.23, *P* = 0.22, Fig. 4) and in the European green crab (*t* = 1.58, *P* = 0.12, Fig. 5). In contrast for the Asian shore crab, we found that the residual external gut markings increased with the residual gut size (parameter estimate = 0.35 ± 0.08, *t* = 4.24, *P* < 0.0001, Fig. 6). Similarly, we also found that the residual external gut markings increased with residual gut size for adult male (parameter estimate = 0.66 ± 0.14, *t* = 4.67, *P* < 0.0001, Fig. 7A), adult female (parameter estimate = 1.07 ± 0.20, *t* = 5.39, *P* < 0.0001, Fig. 7B), and juvenile Atlantic blue crabs (parameter estimate = 0.56 ± 0.10, *t* = 5.50, *P* < 0.0001, Fig. 7C).

## Discussion

Using field collections and publicly available images, we have shown that independent of body size, percent herbivory in the diet increases non-linearly across families of brachyuran crab with the externally measured estimate of the gut width:carapace width ratio. Additionally, the ability of external standardized gut width to predict percent herbivory remained even after accounting for carapace shape that differed phylogentically. We further demonstrated that variation in externally measured gut markings were positively related to variation in gut widths across individuals in some species of brachyuran crabs (Asian shore crabs and Atlantic blue crabs), though not all (European green crabs and mangrove tree crabs). These results suggest that the external carapace markings can be used to estimate percent herbivory to eliminate the need for dissection in some species. Combining results here connecting external carapace markings to gut size with previous work on individual gut size and diet strategies (Griffen & Mosblack, 2011) suggests that external gut markings may even be used in some species to estimate variation in individual diet quality. However, the relationship between diet and external gut markings is not equally clear for all species and additional data are needed to connect external markings to diets of individual organisms. Below we examine two factors, intraspecific diet variation and morphological constraints, that influence the utility of this fast, nonlethal approach to estimating diet. Furthermore, these factors have implications for the evolution of crabs and for morphophysiological tradeoffs in general.

### Intraspecific diet variation as a constraint to using external markings at the species level

Our ability to detect a relationship between crab diet and gut size across species (Fig. 3) based on external gut markings, despite complicating factors, demonstrates the strength of the diet-gut size relationship. Several factors likely increased variation in these two metrics. First, intraspecific diet variation and specialization is common across a broad range of organisms (Bolnick et al., 2003), including crabs (*e.g*., Griffen et al., 2012; Hostert, Pintor & Byers, 2019). Intraspecific diet variation in crabs is caused by ontogenetic changes (*e.g*., Perez & Bellwood, 1988; Stoner & Buchanan, 1990; Lim, Yong & Christy, 2016), site-specific differences (*e.g*., Morrow, Bell & Tewfik, 2014; Griffen et al., 2020b) or seasonal differences (*e.g*., Kennish, 1997; Griffen et al., 2012). Our diet estimates often represent location- and site-specific diet estimates. Second, diets and gut sizes here reflect mean values with very different sample sizes. Diet estimates reflect mean values from a large number of individuals (sometimes thousands), while gut size estimates are means from (usually) five photos available for each species (Table S1). Outliers in the mean values for these two metrics would therefore have influenced gut size estimates much more than diet estimates, due to the smaller sample size for guts. Outliers may have occurred because of the age, location, or timing of collection of individual crabs. Finally, diet variation through time and across sites means that pictures of crabs from which gut size was measured may have had entirely different diets from crabs sampled for diet estimates in published studies. Despite these potential sources of variation that could have obscured the relationship between diet and gut size, we found a strong relationship between these two metrics, even when measured in different individuals, and even when gut size was measured externally, demonstrating the strength of the relationship between diet and gut size.

Ultimately, additional research is needed to assess the ability to determine individual diet variation across conspecifics using external gut size measurements and to examine the extent to which correlations between gut size and external gut markings exist for both males and females, especially when differences in diet exist between the sexes. Given the tight correlation between gut size and external gut size estimates in Atlantic blue crabs shown here, this research may most profitably be conducted (at least initially) on this species or a congener. The reason for the stronger relationship in Atlantic blue crabs compared to the other species examined is unclear, but it may be related to the relatively large body size of this species that provides increased space, and therefore increased flexibility in space use, inside the carapace (see below).

### Morphological constraints

The relationship between diet and external gut markings was influenced by differences in the shape of the carapace across crab families. However, we found that even after controlling for this by using both crab phylogeny (random effect) and using the carapace shape (fixed effect) in our analysis, that standardized gut width still had a strong residual correlation with percent herbivory. Our results suggest that gut size, and possibly even carapace shape, are evolutionarily driven by dietary needs, as diet quality determines gut capacity needs and the shape of the carapace influences the space inside the animal available for the gut and other organs.

Some of the families examined here (*e.g*., *Panopeidae*, *Grapsidae*) showed little variation in gut size, with individual species within a family clustering along the x-axis (gut width:carapace width ratio) in Fig. 3. This suggests that within these families, the gut size for any given size crab may be constrained by shared evolutionary relationships and possibly by the needs of other organs for space within the carapace. Potential evolutionary constraints on gut size should likely limit maximum gut size more than minimum gut size because of the limited space inside the carapace and the need to share that space with other vital organs.

Previous work documents variation in gill, ovary, and hepatopancreas size related to the ecology, lifestyle, season, and dietary strategy of crabs. For instance, gill size is larger for more active species, and relative gill size generally increases moving from land crabs, to intertidal species, to wholly aquatic species (Gray, 1957). Similarly, ovary and hepatopancreas size vary with season (Kennish, 1997) and with the amount and quality of dietary intake (Griffen, 2014). Given the hard exoskeleton of crabs, the internal cavity is constrained and increases in the size of the stomach, gills, ovary, or hepatopancreas are limited by the internal volume of the carapace. When this internal volume is maximally used, which is most likely to occur in crabs towards the end of the molt cycle when they have grown to fill this internal space, growth in any single organ can only come at the expense of a tradeoff in the size of one or more other organs.

Differences in carapace shape across phylogenetic families influence this internal volume and thereby the tradeoffs that must occur in organ size. Hines (1982) examined reproduction across seven families of brachyuran crabs and noted the allometric constraints on reproductive output that can occur because of space limitation inside the carapace. While most species appear to deal with this space limitation by increasing the number of broods produced per year (*i.e*., spreading reproductive allocation through time rather than through space inside the carapace), a limited number of species appear to address this limitation by creatively repositioning the ovary outside of the cephalothorax and into the abdomen, and reducing carapace calcification to enable distension of the body as eggs are produced (Hines, 1992).

These arguments all suggest that there is an upper limit to the amount of internal space that can be devoted to growth of the cardiac stomach. As diet quality decreases, this suggests a maximum gut size and a maximum gut width:carapace width ratio. Based on the data shown in Fig. 3, this maximum ratio would seem to be around 0.6 for most families of crab. The exact magnitude of this ratio depends on family-specific carapace shape. Assuming the same carapace depth (dorsal to ventral distance), a square carapace will maximize the internal volume for a given carapace width, while a more oval-shaped or rounded carapace will have smaller volume for a given carapace width. This reasoning is consistent with maximum ratios for different families shown in Fig. 3, where those with more rounded or oblong carapaces (*Cancridae*, *Portunidae*, *Panopeidae*) have smaller maximum ratios, while those with relatively square carapaces (*Grapsidae*, *Sesarmidae*, *Varunidae*) have much higher maximum ratios. Further research is needed to understand the extent to which carapace shape limits gut size and thus diet, or conversely, the extent to which diet strategies that require a larger gut size has pushed the evolution of a carapace shape that maximizes volume.

The use of external markings to assess the diet of crabs using the gut width to carapace width ratio is not useful for all families of crabs. For example, all the families chosen for this study have carapace shapes where the carapace width is greater than the carapace length. For elongated species, such as those in the superfamily Majoidea and others, these methods may be much less successful. These crabs appear to extend gut size by altering gut length rather than gut width. Thus, while the correlation between gut capacity and diet quality likely still applies to these crabs, it is not possible to accurately assess this using the methods demonstrated here. Additionally, these methods cannot be applied to crabs that have smooth carapaces that lack external gut markings. For instance, many species in the family Ocypodidae have no external gut markings. Despite these limitations, the methods demonstrated here are applicable to a wide range of crab families and should therefore be broadly useful.

In summary, some scientific questions can only be answered by sacrificing study animals, but this is not the case for all research. When highly specific information is needed on dietary consumption, more expensive and time-consuming methods (stable isotopes, gut dissections) will be required. But when rough approximations of diet quality will suffice (*i.e*., percent herbivory), the method outlined here presents a quick, free, nonlethal alternative with several applications. For example, given the highly invasive nature of crabs (Galil, 2008), this method could be used to quickly compare broad diet strategies of invasive crabs in their native and invasive ranges. It could also be used to assess seasonal or regional differences in diet strategies of a species throughout its range. Or it could be useful to assess dietary changes of a species across sites with different levels of human impact. This tool could be further honed by additional research to understand the rate of change of gut size following diet shifts by individual animals, and to more fully clarify the specific dietary characteristics (*i.e*., specific nutrient content, energy content, *etc*.) that influence gut size.

## Supplemental Information

10.7717/peerj.15224/supp-1 Supplemental Information 1 List of online pictures used to assess the relationship between diet and external gut markings across species. Columns include phylogenetic family, genus, and species (used in statistical analysis to control for phylogenetic relationships), as well as sampling location for diet analysis, percent herbivory (rounded to the nearest percent), the literature reference for the publication from which the diet estimate was obtained, the measured gut width (GW) from external markings using the online photo (unitless value), the carapace width (CW) from online photos (unitless value), the GW:CW ratio, the URL where each photo was accessed, and the date that it was accessed.

10.7717/peerj.15224/supp-2 Supplemental Information 2 *Hemigrapsus sanguineus* data used for intraspecific comparisons.

10.7717/peerj.15224/supp-3 Supplemental Information 3 *Carcinus maenas* data used for intraspecific comparisons.

10.7717/peerj.15224/supp-4 Supplemental Information 4 *Callinectes sapidus* data used for intraspecific comparisons.

10.7717/peerj.15224/supp-5 Supplemental Information 5 *Aratus pisonii* data used for intraspecific comparisons.

## Additional Information and Declarations

### Data Availability

The following information was supplied regarding data availability: The raw data are available in the Supplemental Files.
